# Long-term follow-up of cytogenetically normal *CEBPA*-mutated AML

**DOI:** 10.1186/s13045-014-0055-7

**Published:** 2014-09-10

**Authors:** Friederike Pastore, Daniela Kling, Eva Hoster, Annika Dufour, Nikola P Konstandin, Stephanie Schneider, Maria C Sauerland, Wolfgang E Berdel, Thomas Buechner, Bernhard Woermann, Jan Braess, Wolfgang Hiddemann, Karsten Spiekermann

**Affiliations:** Laboratory for Leukemia Diagnostics, Department of Internal Medicine III, University Hospital Munich Grosshadern, Munich, Germany; German Cancer Consortium (DKTK), Heidelberg, Germany; German Cancer Research Center (DKFZ), Heidelberg, Germany; Department of Medical Statistics, Biometry and Epidemiology, University Munich, Munich, Germany; Institute of Biostatistics and Clinical Research, University of Muenster Germany, Muenster, Germany; Department of Medicine A, Hematology and Oncology, University of Muenster, Muenster, Germany; German Society of Hematology and Oncology, Berlin, Germany; Department of Oncology and Hematology, Klinikum Barmherzige Brüder, Regensburg, Germany

**Keywords:** Acute Myeloid Leukemia, Cytogenetically normal AML, Monoallelic CEBPA mutation, Biallelic CEBPA mutation

## Abstract

**Background:**

The aim of this study was to analyze the long-term survival of AML patients with *CEBPA* mutations.

**Patients and methods:**

We investigated 88 AML patients with a median age of 61 years and (1) cytogenetically normal AML (CN-AML), (2) monoallelic (mo*CEBPA*) or biallelic (bi*CEBPA*) *CEBPA* mutation, and (3) intensive induction treatment. 60/88 patients have been described previously with a shorter follow-up.

**Results:**

Median follow-up time was 9.8 years (95% CI: 9.4-10.1 years) compared to 3.2 and 5.2 years in our former analyses. Patients with bi*CEBPA* mutations survived significantly longer compared to those with mo*CEBPA* (median overall survival (OS) 9.6 years vs. 1.7 years, p = 0.008)**.** Patients ≤ 60 years and bi*CEBPA* mutations showed a favorable prognosis with a 10-year OS rate of 81%.

Both, bi- and mo*CEBPA-*mutated groups had a low early death (d60) rate of 7% and 9%, respectively. Complete remission (CR) rates for bi*CEBPA*- and mo*CEBPA-*mutated patients were 82% vs. 70% (p = 0.17). bi*CEBPA-*mutated patients showed a longer relapse free survival (RFS) (median RFS 9.4 years vs. 1.5 years, p = 0.021) and a lower cumulative incidence of relapse (CIR) compared to mo*CEBPA-*mutated patients. These differences in OS and RFS were confirmed after adjustment for known clinical and molecular prognostic factors.

**Conclusions:**

In this long-term observation we confirmed the favorable prognostic outcome of patients with bi*CEBPA* mutations compared to mo*CEBPA-*mutated CN-AML. The high probability of OS (81%) in younger patients is helpful to guide intensity of postremission therapy.

**Electronic supplementary material:**

The online version of this article (doi:10.1186/s13045-014-0055-7) contains supplementary material, which is available to authorized users.

## Background

According to the current recommendations of the WHO [1] the large and heterogeneous group of cytogenetically normal AML (CN-AML) is further stratified by the presence or absence of internal tandem duplications of fms-related tyrosine kinase 3 (*FLT3*-ITD), mutations of nucleophosmin (*NPM1*) and mutations in the CCAAT/enhancer binding protein (C/EBP) alpha (*CEBPA*). In fact, “AML with mutated *CEBPA”* has been classified as its own category in the current WHO classification [1].

The gene encoding for the CCAAT/enhancer binding protein-α (*CEBPA*) is located on chromosome 19 band q13.11. It was first full-length cloned in 1997 [2]. The *CEBPA* protein is 42 kDa of size. It is expressed in myelomonocytic cells and upregulated in granulocytic differentiation acting as a myeloid transcription factor. Mutations of *CEBPA* in AML were first described in 2001 [3]. N-terminal frameshift mutations lead to the overexpression of a truncated 30 kDA isoform of *CEBPA* that suppresses *CEBPA* function in a dominant negative way. C-terminal mutations occur mainly in the basic Zipper (bZIP) domain of *CEBPA*, and impair its function to homodimerize and heterodimerize with other proteins as well as its DNA binding [4]. *CEBPA* knock-out mice show a selective block of differentiation lacking mature granulocytes while other hematopoietic cells differentiate regularly [5]. Mutations of *CEBPA* have been shown to be associated with CN-AML where they occur with frequencies of 8 -18% [6–10] and with the French-American-British (FAB) subtypes M1 and M2 [11].

*CEBPA* mutations can occur as monoallelic mutations (mo*CEBPA*) or as biallelic mutations (bi*CEBPA*). Patients with bi*CEBPA* mutations usually have a C-terminal mutation on one allele and an N-terminal mutation on the other allele, resulting in a lack of *CEBPA* wildtype allele expression [12,13]. We and others have reported earlier that the positive prognostic impact on outcome is restricted to patients with bi*CEBPA* mutations [6,13–16]. Except for the studies of Taskesen [16] and Green [14] patient numbers with mutated *CEBPA* at diagnosis were small (n < 50) and median follow-up times were <10 years in the majority of the upper mentioned analyses.

The objective of this study was to investigate if the effect of mo*CEBPA* versus (vs.) bi*CEBPA* mutations on outcome was true also in a longer follow-up period and to elucidate the clinical course of disease in bi*CEBPA* mutations patients.

## Methods

### Patients

In this analysis we included all cytogenetically normal (CN) AML patients with a monoallelic or a biallelic *CEBPA* mutation treated within the two large multicenter AML Cooperative group clinical studies, the AMLCG99 trial [NCT00266136] and the AMLCG2008 trial [NCT01382147; EUDRACT2007-003103-12] (randomization from July 1999 until December 2012; approved by the local institutional review boards) or in analogy to clinical studies (treatment start from April 2000 until March 2013) in our university hospital. We identified 88 patients fulfilling these criteria. A subset of 60 of these patients have been investigated in previous publications with a shorter follow-up [6,17].

Clinical parameters available at first diagnosis included age, sex, Eastern Cooperative Group (ECOG) performance status [18], the French-American-British (FAB) morphologic AML subtype, the origin of AML (de novo vs. secondary or therapy-related AML), white blood cell count (WBC), platelet count, haemoglobin level, lactate dehydrogenase (LDH) level, myeloid blasts in the bone marrow (BM) and in the peripheral blood (PB).

All patients were treated with intensive induction chemotherapy. 76 patients (86%) were treated within the AMLCG99 (n = 68) and AMLCG2008 (n = 7) and the HD98-A study (n = 1). Details of the AMLCG99 study and AMLCG2008 study have been published before [19–21]. 12 patients (14%) were treated in analogy to the AMLCG studies or with a classical “7 + 3” therapeutic regimen. The analysis included 19 patients who underwent allogeneic stem cell transplantation (SCT): as consolidation in first CR (n = 7; 8% of patients), at the time of relapse (n = 8; 9.1%) or primary refractory disease (n = 4; 4.5%).The studies were approved by the ethics committees of all participating institutions.

### Cytogenetic and molecular analysis

Cytogenetic and molecular analyses were performed on BM aspirates. For cytogenetic analyses ≥ 20 metaphases were required. AML were classified as cytogenetically normal according to the guidelines of the international system of cytogenetic nomenclature (ISCN) [22]. Mutations of *NPM1* [23], *FLT3*-ITD [24,25], *FLT3*-TKD [26], mo*CEBPA* and bi*CEBPA* [6,10] and *MLL*-PTD [27] were analyzed as previously published.

### Statistical analysis

The outcome parameter overall survival (OS) was calculated from the date of first diagnosis to death. Relapse-free-survival (RFS) was assessed in all patients having achieved a complete remission (CR) or a CR with incomplete recovery (CRi) according to the standard guidelines of the ELN [28] and was calculated from the date of CR/CRi until relapse or death. For patients who underwent allogeneic SCT OS and RFS times were censored at the date of allogeneic transplantation.

For pairwise comparisons of dichotomous parameters between mo*CEBPA* and bi*CEBPA*-mutated patients the *χ*^2^-test/Fisher’s exact test was applied. The Mann–Whitney *U* test was performed for the comparison of continuous parameters between mo*CEBPA* and bi*CEBPA*-mutated patients.

Comparisons of OS and RFS between patients with mo*CEBPA* and bi*CEBPA* mutations were obtained applying the Kaplan Meier method and the log rank test. Median follow-up was calculated with the reversed Kaplan Meier method. Univariable and multivariable Cox regression analyses were performed for OS and RFS to adjust for potential imbalances of known clinical and molecular prognostic factors summarized in the PINA [29]. To evaluate the effect of bi*CEBPA* vs. mo*CEBPA* mutations on AML-specific survival taking into account only deaths related to AML, a competing risk analysis was performed treating death unrelated to AML, and allogeneic SCT as competing events. Likewise, the cumulative incidence of relapse (CIR) was calculated for all patients in CR/CRi treating death in CR and allogeneic SCT in first CR as competing events. Cumulative incidence rates [30] and hazard ratios (HR) [31] between the risk groups were calculated and compared by the Gray test [32]. The comparison of OS in bi*CEBPA-* vs. mo*CEBPA-*mutated patients was tested with a significance level of 5%. All other p-values are descriptive. Analyses were performed using SPSS software, version 20.0 (SPSS, Chicago, IL) and the R 3.0.1 software package (R foundation for statistical computing, Vienna, Austria).

## Results

### Comparison of mo*CEBPA*- vs. bi*CEBPA*-mutated patients with respect to clinical and molecular parameters

Analyses were performed in 88 patients with CN-AML and a mutation of *CEBPA*. Median age was 61 years, the majority (86%) had de novo AML and an ECOG performance status of 0–2 (96%). 45 patients showed a bi*CEBPA* mutation, 43 patients had a mo*CEBPA* mutation. Mutations of *NPM1* and *FLT3*-TKD were present in 19% and 6%, *FLT3*-ITD and *MLL*-PTD occurred in 25% and 1% of patients (Additional file 1: Table S1). 67% of patients received a double induction therapy (Table 1). Median follow-up time was 9.8 years (95% CI: 9.4-10.1). 4.5% of patients died within 30 days after start of therapy. Median OS was 3.0 years (95% CI: 0.9-5.2) and median RFS in 67 patients who have achieved a CR/CRi was 2.3 years (95% CI: 1.0-3.7) (Table 1). 28 (42%) of patients in CR/CRi relapsed and 51 (58%) of patients died.

**Table 1:** **Therapy and outcome**

	**All patients (N = 88)**		**mo** ***CEBPA*** **(N = 43)**		**bi** ***CEBPA*** **(N = 45)**		***P***
	**N**	**%**	**N**	**%**	**N**	**%**	
**Number of induction cycles**							
1	29	33	16	37	13	30	0.41
2	59	67	27	63	32	71	
**Induction therapy regimen**							0.48
In study	76	86	36	84	40	89	
In analogy to study	12	14	7	16	5	11	
**Allogeneic SCT**	19	22	10	23	9	20	0.75
in 1.CR	7	8	3	7	4	9	
Primary refractory	4	5	3	7	1	2	
At relapse	8	9	4	9	4	9	
**Early death until day 30**	4	5	3	7	1	2	0.28
**Early death until day 60**	7	8	4	9	3	7	0.65
CR/CRi*	67	76	30	70	37	82	0.17
**Median OS**, 95% CI, years	3.0 (0.9-5.2)		1.7 (0.7-2.7)		9.6 (NA)		0.008
**Median RFS**, 95% CI, years	2.3 (1.0-3.7)		1.5 (0.4-2.5)		9.4 (NA)		0.021

*62 patients have achieved a CR; 5 patients have achieved a CRi;

Abbreviations: bi*CEBPA* biallelic mutation in the CCAAT/enhancer-binding protein alpha, *CR* complete remission, *CRi* CR with incomplete revovery, mo*CEBPA* monoallelic mutation in the CCAAT/enhancer-binding protein alpha, *N* number, *NA* not applicable, *SCT* stem cell transplantation.

There were less female patients in the bi*CEBPA* group compared to the mo*CEBPA* group (44% vs. 72%) (Additional file 1: Table S1)**.** The presence of bi*CEBPA* mutations was associated with a higher rate of de novo AML, a higher hemoglobin level, lower platelet count and lower frequencies of additional mutations of *NPM1*, *FLT3*-TKD or the presence of *FLT3*-ITD. All other clinical and molecular parameters as well as therapeutic regimen including allogeneic SCT were evenly distributed between patients with mo*CEBPA* and bi*CEBPA* mutations (Table 1).

### bi*CEBPA*-mutated patients show a longer OS, RFS and a lower CIR

Early death rates at day 30 and at day 60 were not different between patients with bi*CEBPA* and mo*CEBPA* mutations (Table 1).

**Figure 1:**
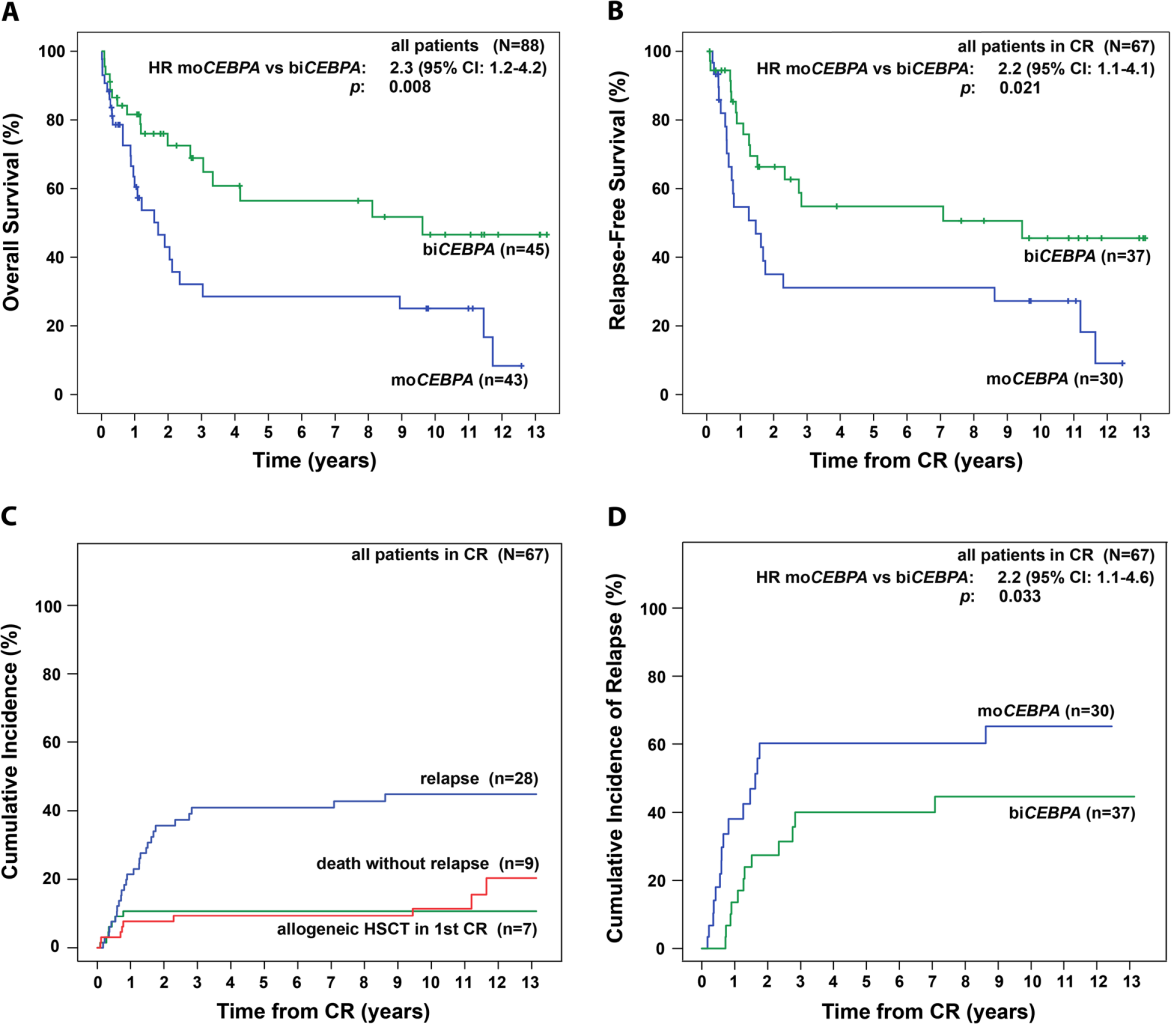
**Outcome in patients with bi** ***CEBPA*** **mutations compared to mo** ***CEBPA*** **mutations. (A)** OS in all patients **(B)** RFS in all patients in CR **(C)** Cumulative incidence of relapse, death without relapse and allogeneic transplantation in 67 patients with a CR and **(D)** cumulative incidence of relapse in mo*CEBPA* or bi*CEBPA-*mutated patients. Abbreviations: bi*CEBPA*, biallelic mutation in the CCAAT/enhancer-binding protein alpha; *CI*, confidence interval; *CR*, complete remission; mo*CEBPA*, monoallelic mutation in the CCAAT/enhancer-binding protein alpha.

**Figure 2:**
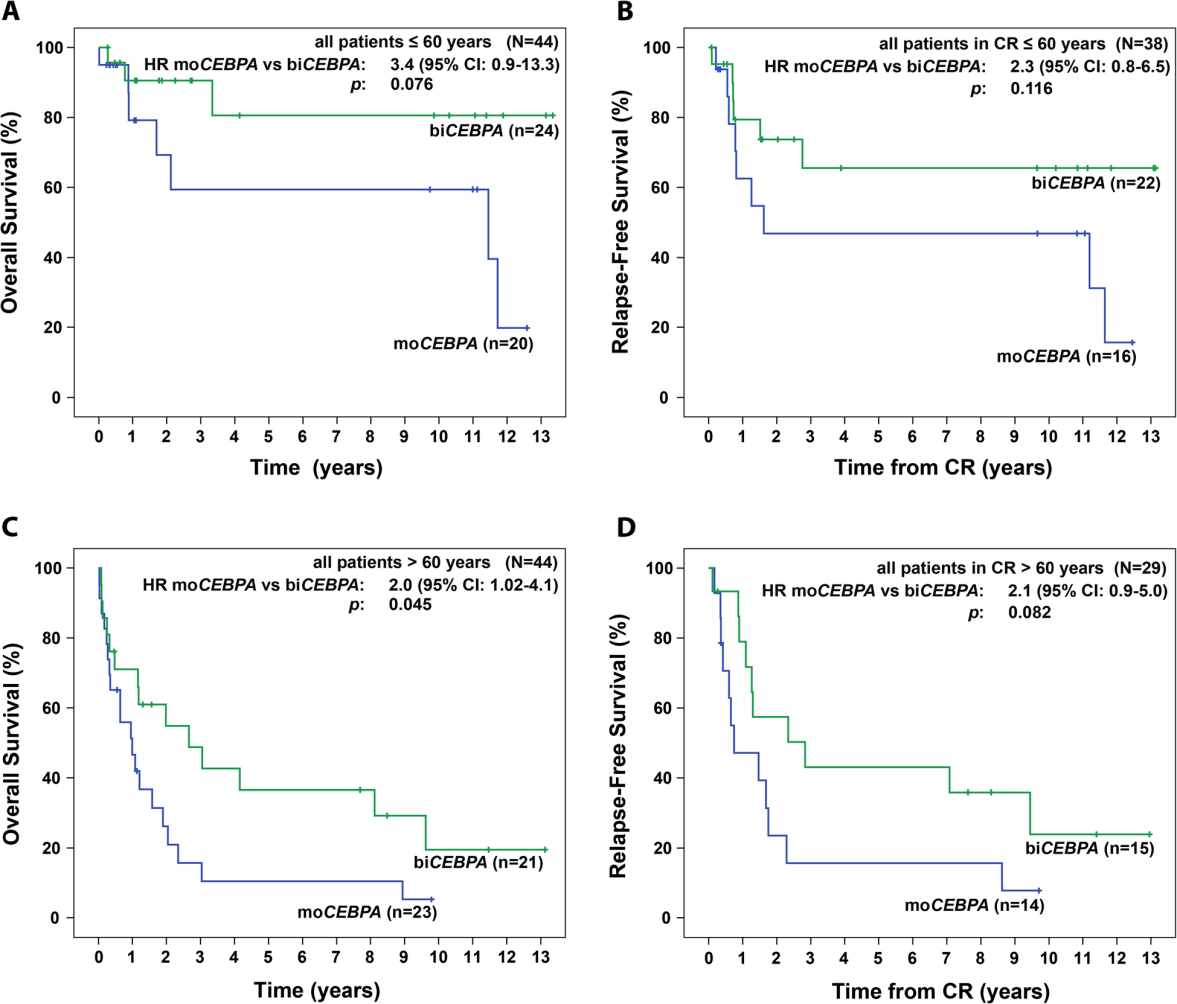
**OS and RFS in patients ≤/>60 years of age with bi** ***CEBPA*** **mutations compared to mo** ***CEBPA*** **mutations. (A)** OS in patients ≤ 60 years **(B)** RFS in patients in CR ≤ 60 years **(C)** OS in patients > 60 years **(D)** RFS in patients in CR > 60 years. Abbreviations: bi*CEBPA*, biallelic mutation in the CCAAT/enhancer-binding protein alpha; CI, confidence interval; CR, complete remission; mo*CEBPA*, monoallelic mutation in the CCAAT/enhancer-binding protein alpha.

In accordance with previous results, patients with bi*CEBPA* mutations survived significantly longer compared to those with a mo*CEBPA* mutations (median OS 9.6 years vs. 1.7 years, p = 0.008) (Figure 1A). This survival benefit was also evident in patients > 60 years (bi*CEBPA* vs. mo*CEBPA*: 5-years OS: 37% vs. 11%, 10-years OS: 20% vs. 5%, p = 0.045; (Figure 2C)) and by trend in patients ≤ 60 years (bi*CEBPA* vs. mo*CEBPA*: 5-years OS and 10-years OS: 81% vs. 59%, p = 0.076 (Figure 2A)).

Consistent with the long follow-up, nine patients died in CR of causes unrelated to AML. Results of competing risk analyses treating death related to AML and death unrelated to AML as competing are illustrated in the supplement (Additional file 1: Figure S1A). bi*CEBPA-*mutated patients displayed a lower cumulative incidence of death related to AML compared to mo*CEBPA-*mutated patients (p = 0.028) (Additional file 1: Figure S1B).

37 patients (82%) with bi*CEBPA* mutations and 30 patients (70%) with mo*CEBPA* mutations achieved a CR/CRi (p = 0.17) (Table 1). bi*CEBPA-*mutated patients displayed a longer RFS compared to mo*CEBPA-*mutated patients (median RFS 9.4 years vs. 1.5 years, p = 0.021) (Figure 1B, Table 1). A trend to a longer RFS in bi*CEBPA*-mutated patients was seen in subgroups ≤ 60 and > 60 years of age (p = 0.116 and p = 0.082, respectively) (Figure 2B and D).

CIR rates were lower in patients carrying bi*CEBPA* compared to mo*CEBPA* mutations (5-year CIR rates: 40% vs. 60%, respectively; 10-year CIR rates: 45 vs. 65%, respectively; p = 0.036) (Figure 1C,D). Median time from CR to relapse was longer in the bi*CEBPA*-mutated cohort vs. the mo*CEBPA*-mutated patients (not reached vs. 1.6 years, p = 0.033). In the mo*CEBPA*-mutated patients, 63% relapsed within the first and 94% within two years. In contrast, bi*CEBPA*-mutated patients appeared to relapse later during follow-up: only 33% relapsed in the first year, 67% within the second year and 92% within the first three years. In both cohorts, the majority of patient relapsed in the first three years. Two patients - one with a bi*CEBPA* and one with a mo*CEBPA* mutation - showed late relapses after 7 and 8 years. In both cases, we do not have diagnostic bone marrow aspirates to verify if these patients show the same cytogenetic and mutational profile as at diagnosis. Due to the latency of many years and the preceding chemo- and/or radiation therapy, we suspect that these AML relapses might be therapy-associated AML.

A detailed description of relapse and survival after achievement of a CR according to therapy has been provided in the Additional file 1.

### Adjustment for co-occurring mutations and clinical prognostic factors

Since *FLT3*-ITD and *FLT3*-TKD and *NPM1* mutations, were more common in mo*CEBPA-*mutated patients, we performed multivariable analyses to adjust for potential confounding effects. The positive impact of bi*CEBPA* vs. mo*CEBPA* mutations on outcome was confirmed when adjusting for co-occurring *FLT3*-ITD and *FLT3*-TKD mutations, *NPM1* mutations, the PINA_OS_ and PINA_RFS_ [29] scores without bi*CEBPA* mutations (Table 2) and in analyses with *FLT3*-wildtype patients only (Additional file 1: Figure S2).

**Table 2:** **Cox Regression adjusted for additional markers**

	**OS**			**RFS**		
	**HR**	**95% CI**	**p**	**HR**	**95% CI**	**p**
bi*CEBPA* versus mo*CEBPA* (univariable analysis)	0.4	0.2-0.8	0.008	0.5	0.2-0.9	0.021
bi*CEBPA* versus mo*CEBPA* (adjusted for *FLT3*-ITD)	0.4	0.2-0.8	0.006	0.5	0.2-0.9	0.021
bi*CEBPA* versus mo*CEBPA* (adjusted for *FLT3*-TKD)	0.4	0.2-0.8	0.012	0.4	0.2-0.9	0.018
bi*CEBPA* versus mo*CEBPA* (adjusted for *FLT3*-ITD and *FLT3*-TKD)	0.4	0.2-0.8	0.006	0.4	0.2-0.8	0.012
bi*CEBPA* versus mo*CEBPA* (adjusted for *FLT3*-ITD, *NPM1* mutations and interaction *NPM1/FLT3*-ITD)	0.2	0.1-0.5	<0.001	0.2	0.1-0.6	0.003
bi*CEBPA* versus mo*CEBPA* (adjusted for *NPM1* mutations)	0.2	0.1-0.5	<0.001	0.2	0.1-0.6	0.002
bi*CEBPA* versus mo*CEBPA* (adjusted for PINA_OS_ or PINA_RFS_ (both without bi*CEBPA*))	0.2	0.1-0.4	<0.001	0.2	0.1-0.6	0.001

Abbreviations: bi*CEBPA* biallelic mutation in the CCAAT/enhancer-binding protein alpha, *CI* confidence interval, *FLT3*-ITD internal tandem duplication of the *FLT3* gene, *FLT3*-TKD mutation in the tyrosine kinase domain of the *FLT3* gene, *HR* hazard ratio, interaction *NPM1*/*FLT3*-ITD, *NPM1* positive/*FLT3*-ITD positive versus *NPM1* negative or *FLT3*-ITD negative, mo*CEBPA* monoallelic mutation in the CCAAT/enhancer-binding protein alpha, *NPM1*, nucleophosmin gene, *OS* Overall survival, *p* p value, *PINA*_*OS*_ Prognostic Index for OS in cytogenetically normal AML [29], *PINA*_*RFS*_ Prognostic Index for RFS in cytogenetically normal AML [29], *RFS* Relapse-free survival.

### Relapsed patients with bi*CEBPA* and mo*CEBPA* mutations show similar outcomes

Treatment modalities at relapses (palliative vs. intensive treatment; allogeneic SCT) were not different between mo*CEBPA-* and bi*CEBPA*-mutated patients. Both *CEBPA* cohorts, included similar amounts of patients ≤ 60 years (mo*CEBPA*: 6/11; bi*CEBPA* 4/9) and > 60 years (mo*CEBPA*: 5/11; bi*CEBPA* 5/9). Although, intensively treated bi*CEBPA*-mutated patients showed a tendency to a higher second CR rate compared to mo*CEBPA-*mutated patients (78% vs. 45%, p = 0.142) survival after relapse was not different between patients with mo*CEBPA* or bi*CEBPA* mutations (data not shown). In both cohorts, long-term survival after relapse was only possible for patients treated with allogeneic SCT. A detailed description of treatment at relapse, achievement of a second CR and survival is given in the Additional file 1.

## Discussion

According to the current WHO classification and the ELN guidelines patients with mutated *CEBPA* represent a cohort with a favorable prognosis [1,28]. We and others have shown before, that this favorable prognostic effect is restricted to the group of patients with bi*CEBPA*, in contrast to mo*CEBPA* [6] mutations. The aim of our current analysis was to test if the favorable prognostic effect of bi*CEBPA* mutations was still evident within a (1) larger patient cohort and (2) after a longer follow-up period.

Due to the low frequency of *CEBPA* mutations of about 8 - 18% in CN-AML, studies are often based on small patient numbers [6,13,15], which reduces statistical power. Our study includes 88 patients, 75 of whom were treated homogeneously within AMLCG99 and AMLCG2008 trials.

Except for the study of Green et al. [14] with a medium follow-up for survivors of 11.7 years, median follow-up in the published literature including our previous publication is mostly ≤ 5 years [6,13,15,16,33]. The present analysis has a long medium follow-up of almost 10 years (9.8 years) and a maximum follow-up of 13.3 years. This allowed to detect late relapses.

In accordance with previous analyses [13–15], bi*CEBPA*-mutated AML patients displayed a significantly longer OS compared to mo*CEBPA*-mutated patients. Differences in OS might be caused by differences in early death rate, achievement of a CR and RFS/incidence of relapses. Patients with a bi*CEBPA* mutation showed a slightly lower early death rate at day 60 (6.7% versus 9.3%, Table 1) and a higher CR rate (CR rates: 82% versus 70%, p = 0.17, respectively) compared to patients with a mo*CEBPA* mutation. Interestingly, overall early death rate until day 60 in all *CEBPA*-mutated patients in our cohort was 8.0%, which is lower compared to data in the literature of 10% to 16% [19,20,34]. These differences might be caused by the fact that these studies included all cytogenetic risk groups and not only CN-AML patients. Most importantly, we could clearly see a better RFS in bi*CEBPA*-mutated patients. This improved RFS was also seen by others [14,15]. Moreover, we could demonstrate a lower CIR in bi*CEBPA*-mutated compared to mo*CEBPA-*mutated patients. However, to what extent a lower early death rate, a higher CR rate or a longer RFS caused the prolonged survival still remains open and needs to be investigated in larger patient cohorts.

The favorable prognosis of bi*CEBPA*-mutated AML was most evident in patients ≤ 60 years who displayed a 10-years OS of 81% and a 10-years RFS of 66%.

In contrast to relapse in mo*CEBPA*-mutated patients, occurring in the majority of patients (63%) within the first year after achievement of a CR, only about one third of bi*CEBPA*-mutated patients relapsed within the first year. Almost all mo*CEBPA*-mutated patients and bi*CEBPA*-mutated patients relapsed within the first three years.

Interestingly, one patient with a bi*CEBPA*-mutated AML and one patient with a mo*CEBPA*-mutated AML relapsed more than 7 years after CR. To our knowledge, this is the first case of such a late relapse in bi*CEBPA*-mutated AML. These late AML relapses might be therapy-associated and display different cytogenetics and molecular mutational patterns. Unfortunately, due to lack of material, we cannot confirm the presence of a *CEBPA* mutation at relapse. Both cases underline the importance of a long-term follow-up of patients with AML and *CEBPA* mutations.

The long patient follow-up enabled us to detect patients with late deaths (>5 years) unrelated to AML: Two patients with a mo*CEBPA* mutation and one patient with a bi*CEBPA* mutation died in CR after 11.7 years (cause unknown), 11.5 years (cause: development of a cancer of unknown primary) and 9.6 years (cause: cardiac failure). Thus, the inclusion of “AML unrelated death” helps to better estimate prognosis, especially in elderly co-morbide patients.

Schlenk et al. [35] recently showed in a large cohort of 124 CN-AML patients with bi*CEBPA* mutations in first CR, a significantly longer RFS, but similar OS, for those patients receiving allogeneic or autologous transplantation compared to chemotherapy only. We also found a similar OS in our bi*CEBPA*-mutated patients (n = 4) receiving allogeneic SCT in first CR, compared to those obtaining chemotherapy (n = 30), although we could not detect an effect of allogeneic SCT on RFS, which might be due to the small number of patients receiving allogeneic SCT (data not shown). In accordance with Schlenk et al. [35] we found a high second CR rate after reinduction therapy of 78% in bi*CEBPA-*mutated patients treated with intensive protocols. Due to transplant-related mortality, infections and a high relapse rate after the second CR, this did not result in a longer OS compared to relapsed mo*CEBPA*-mutated patients. These results have to be interpreted with caution since they are limited by small patient numbers. Schlenk et al. [35] furthermore showed that only relapsed patients treated with allogeneic SCT, but not those treated with chemotherapy alone, survived longer than 2 years. Our analyses - although performed in a smaller patient cohort - are in line with these results: In our cohort, patients with bi*CEBPA* mutations that have received allogeneic SCT at the time of relapse are still alive (after 7.0 and 11.3 years) or have died due to treatment related mortality, but not due to AML relapse/refractory AML. In contrast, all 5 patients receiving chemotherapy at the time of relapse died (median OS after relapse: 0.9 years).

## Conclusion

In conclusion, our study with a long-term follow-up of homogeneously treated CN-AML of almost 10 years clearly showed that patients carrying bi*CEBPA* mutations have a substantially better OS and RFS as well as a relevantly lower CIR compared to patients with mo*CEBPA* mutations. The excellent prognosis of younger AML patients with bi*CEBPA* mutations (10-year OS of 81%) might lead to a reduction of the intensity of postremission therapy in this subgroup.

## Consent

Written informed consent was obtained from patients for the data collection, analysis and publication.

## Additional file

Additional file 1:
**Data supplement.**
