# Supplementary material for: Long-term follow-up of cytogenetically normal CEBPA-mutated AML
Source: J Hematol Oncol. 2014 Sep 10;7:55. doi: 10.1186/s13045-014-0055-7 (PMC4172831; doi:10.1186/s13045-014-0055-7)

**Additional file 1** **DATA SUPPLEMENT**

**Long-term follow-up of cytogenetically normal CEBPA-mutated AML**

*Survival and Relapse after achievement of a CR according to therapy*

- *Chemotherapy in 1^st^ CR*

30/37 (81%) of patients with bi*CEBPA* and 21/30 (70%) with mo*CEBPA* mutations received consolidation chemotherapy in first CR. There was a trend to a longer 5-year-survival in bi*CEBPA* versus mo*CEBPA* patients (61.8% versus 35.0%, p=0.051).

Patients with a bi*CEBPA* mutation displayed a trend to a lower relapse rate and longer RFS (37%; 7.1 years) compared to mo*CEBPA*-mutated (52%; 1.7 years), respectively.

- *Allogeneic stem cell transplantation in 1^st^ CR*

Four of the bi*CEBPA* patients underwent allogeneic SCT in first CR. Median survival after allogeneic SCT was 7.3 years (range 0.3-12.3 years). 3/4 are still alive without relapse. One patient died from cerebral toxoplasmosis without recurrence of AML.

Three of the mo*CEBPA* patients underwent allogeneic SCT with a median survival after allogeneic SCT of 8.4 years (range: 7.7-9.4 years).

All three patients are still alive without relapse.

Importantly, no patient with allogeneic SCT in first CR relapsed.

- *No consolidation in 1^st^ CR*

3/45 patients with a bi*CEBPA and* 6/43 patients with a mo*CEBPA* mutation did not receive any further chemotherapy in first CR, due to their poor ECOG performance status.

*Treatment at relapse, achievement of a second CR and survival*

- *Relapsed patients with biCEBPA mutations*

4/12 (33%) patients within the bi*CEBPA*-mutated cohort underwent reinduction chemotherapy with FLAG IDA (Idarubicin, fludarabine, cytarabine, G-CSF) or S-HAI sequential high-dose AraC, idarubicin), followed by allogeneic SCT at the time of relapse. Two of these patients died within 0.5 years after allogeneic SCT due to transplant-related mortality (transplant failure and severe liver failure caused by severe GVHD). The other two patients are still alive and in CR 7.0 and 11.3 years after allogeneic SCT.

All patients treated with chemotherapy without allogeneic SCT (N=5/12 salvage chemotherapy; N=3/12 palliative chemotherapy) died in refractory or in a second relapse of AML within 0.9 years (range: 0.02-1.3 years) after relapse.

- *Relapsed patients with moCEBPA mutation*

4/16 (25%) of patients within the mo*CEBPA*-mutated cohort underwent allogeneic SCT at relapse. Two patients died within 1.3 years after allogeneic SCT due to a second relapse and one died from an infection. The fourth one is still alive in second CR for >8 years.

All patients treated without allogeneic SCT (N=7/16 salvage chemotherapy; N=5/16 palliative chemotherapy) died with a median survival after relapse of 0.2 years (range: 0.01-1.7 years) due to refractory/second relapsed AML (N=9) or infectious complication in CR or aplasia (N=3).

| **TABLE S1 – Baseline characteristics** | | | | | | | | | | | | |
| --- | --- | --- | --- | --- | --- | --- | --- | --- | --- | --- | --- | --- |
|  | **All patients (N=88)** | | |  | **mo*CEBPA* (N=43)** | | |  | **bi*CEBPA* (N=45)** | | | ***P*** |
|  | N % | | |  | N % | | |  | N % | | |  |
| **Age, years** |  |  |  |  |  |  |  |  |  |  |  |  |
| median |  | 61 |  |  |  | 63 |  |  |  | 60 |  | 0.51 |
| range |  | 16-84 |  |  |  | 16-78 |  |  |  | 20-84 |  |  |
| **Female** | 51 |  | 58 |  |  | 31 | 72 |  |  | 20 | 44 | 0.009 |
| **ECOG (n=71)** |  |  |  |  |  |  |  |  |  |  |  |  |
| 0 | 19 |  | 27 |  | 10/35 |  | 29 |  | 9/36 |  | 25 | 0.73 |
| 1 | 35 |  | 49 |  | 15/35 |  | 43 |  | 20/36 |  | 56 |  |
| 2 | 14 |  | 20. |  | 8/35 |  | 23 |  | 6/36 |  | 17 |  |
| 3 | 2 |  | 3 |  | 1/35 |  | 3 |  | 1/36 |  | 3 |  |
| 4 | 1 |  | 1 |  | 1/35 |  | 3 |  | 0/36 |  | 0 |  |
| **FAB type (n=87)** |  |  |  |  |  |  |  |  |  |  |  |  |
| M0 | 1 |  | 1 |  | 0/42 |  | 0 |  | 1 |  | 2 | 0.52 |
| M1 | 32 |  | 37 |  | 16/42 |  | 38 |  | 16 |  | 36 |  |
| M2 | 42 |  | 48 |  | 18/42 |  | 43 |  | 24 |  | 54 |  |
| M4 | 7 |  | 8 |  | 5/42 |  | 12 |  | 2 |  | 4 |  |
| M6 | 5 |  | 6 |  | 3/42 |  | 7 |  | 2 |  | 4 |  |
| M3, M5, M7 | 0 |  | 0 |  | 0/42 |  | 0 |  | 0 |  | 0 |  |
| M1/M2 | 74 |  | 85 |  | 34/42 |  | 81 |  | 40 |  | 89 | 0.30 |
| **Type of disease** |  |  |  |  |  |  |  |  |  |  |  |  |
| De novo AML | 76 |  | 86 |  | 32 |  | 74 |  | 44 |  | 98 | 0.006 |
| sAML | 9 |  | 10 |  | 8 |  | 19 |  | 1 |  | 2 |  |
| tAML | 3 |  | 3 |  | 3 |  | 7 |  | 0 |  | 0 |  |
| **WBC, x 10^9^/L** |  |  |  |  |  |  |  |  |  |  |  |  |
| Median |  | 28.0 |  |  |  | 31.0 |  |  |  | 26.4 |  | 0.96 |
| Range |  | 0.9-379.0 |  |  |  | 1.0-379.0 |  |  |  | 0.9-289.0 |  |  |
| **Platelet count, x 10^9^/L** |  |  |  |  |  |  |  |  |  |  |  |  |
| median |  | 42 |  |  |  | 57 |  |  |  | 34 |  | 0.020 |
| range |  | 3-367 |  |  |  | 3-367 |  |  |  | 3-176 |  |  |
| **Hemoglobin level, g/dl** |  |  |  |  |  |  |  |  |  |  |  |  |
| median |  | 9.5 |  |  |  | 8.9 |  |  |  | 10.0 |  | 0.021 |
| range |  | 2.7-13.8 |  |  |  | 2.7-13.2 |  |  |  | 7.1-13.8 |  |  |
| **Bone marrow blasts, % (n=82)** |  | N=82 |  |  |  | N=38 |  |  |  | N=44 |  |  |
| median |  | 80 |  |  |  | 80 |  |  |  | 72 |  | 0.45 |
| range |  | 20-100 |  |  |  | 20-97 |  |  |  | 20-100 |  |  |
| **Peripheral blasts, % (n=76)** |  | N=76 |  |  |  | N=35 |  |  |  | N=41 |  |  |
| median |  | 61 |  |  |  | 54 |  |  |  | 65 |  | 0.11 |
| range |  | 1-98 |  |  |  | 1-93 |  |  |  | 5-98 |  |  |
| **LDH, U/l (n=75)** |  | N=75 |  |  |  | N=36 |  |  |  | N=39 |  | 0.92 |
| median |  | 471 |  |  |  | 450 |  |  |  | 477 |  |  |
| range |  | 152-3524 |  |  |  | 152-3524 |  |  |  | 205-2510 |  |  |
| ***NPM1* mutated (n=79)** | 15 |  | 19 |  | 15/34 |  | 44 |  | 0 |  | 0 | <0.001 |
| ***FLT3*-ITD** | 22 |  | 25 |  | 16 |  | 37 |  | 6 |  | 13 | 0.010 |
| ***NPM1* mutated/*FLT3*-ITD wildtype (n=79)** | 6 |  | 8 |  | 6/34 |  | 18 |  | 0 |  | 0 | 0.003 |
| ***FLT3*-TKD (n=83)** | 5 |  | 6 |  | 5/39 |  | 13 |  | 0/44 |  | 0 | 0.014 |
| ***MLL*-PTD (n=87)** | 1 |  | 1 |  | 1 |  | 2 |  | 0/44 |  | 0 | 0.31 |

Abbreviations: mo*CEBPA*, monoallelic mutation in the CCAAT/enhancer-binding protein alpha; bi*CEBPA*, biallelic mutation in the CCAAT/enhancer-binding protein alpha; ECOG, performance status according to the Eastern Cooperative Oncology Group; FAB type, immunophenotype according to the French-American-British classification of AML; *FLT3*-ITD, internal tandem duplication of the *FLT3* gene; *FLT3*-TKD, point mutation in the tyrosine kinase domain of the *FLT3* gene; LDH, lactate dehydrogenase; *MLL*-PTD, partial tandem duplication in the *MLL* gene; N, number; *NPM1*, nucleophosmin1; sAML, secondary AML; tAML, therapy-related AML; WBC, white blood count.

**Figure S1: Outcome in patients with bi*CEBPA* mutations compared to mo*CEBPA* mutations (A)** Cumulative incidence of death related to AML, death not related to AML and allogeneic transplantation in all 88 patients **(B)** cumulative incidence of death related to AML in mo*CEBPA* or bi*CEBPA* mutated patients Abbreviations: bi*CEBPA*, biallelic mutation in the CCAAT/enhancer-binding protein alpha; CI, confidence interval; HR, hazard ratio; mo*CEBPA*, monoallelic mutation in the CCAAT/enhancer-binding protein alpha; N, number; n, number; p, p-value; vs, versus.


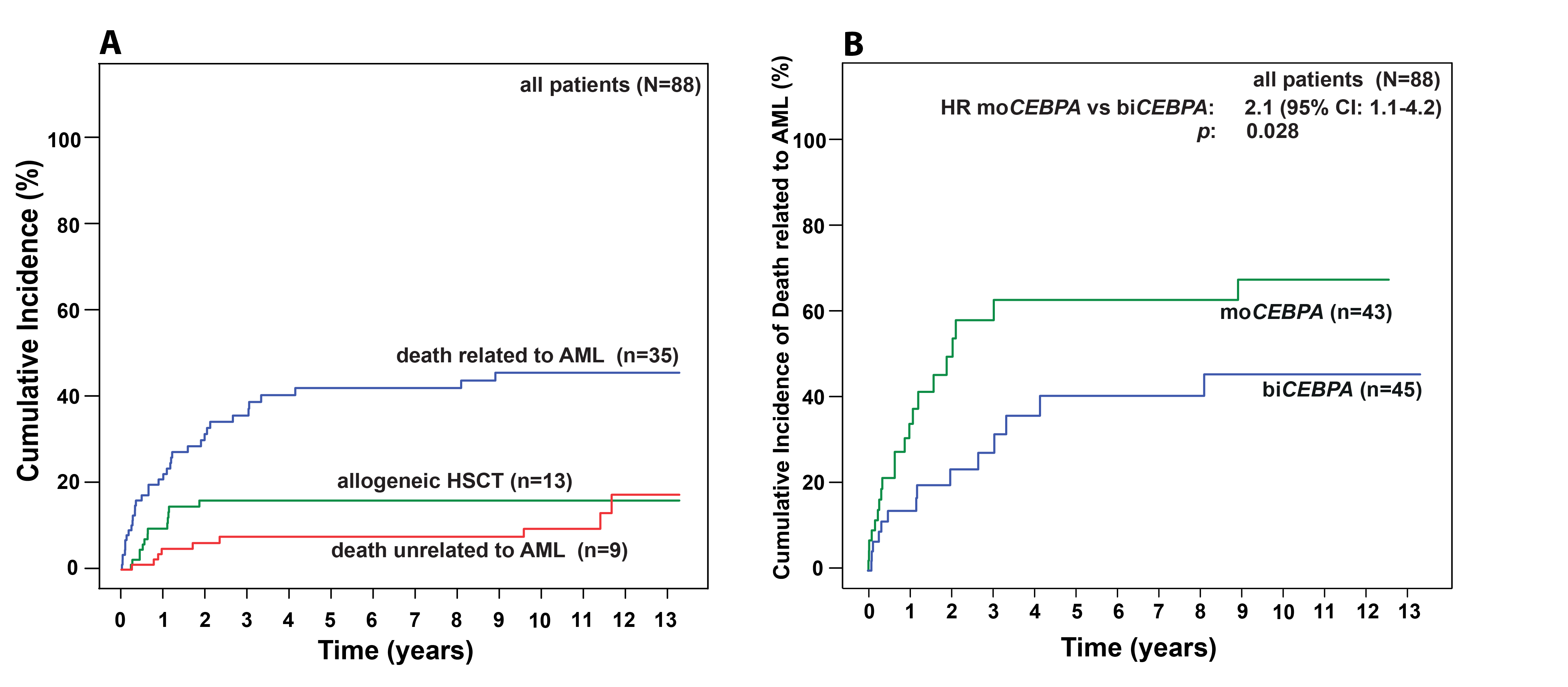


**Figure S2: Outcome in patients with bi*CEBPA* mutations compared to mo*CEBPA* mutations and *FLT3* wild-type (A)** OS in all 58 patients **(B)** RFS in all patients in CR **(C)** Cumulative incidence of relapse, death without relapse and allogeneic transplantation in 44 patients with a CR and **(D)** cumulative incidence of relapse in mo*CEBPA* or bi*CEBPA* mutated patients.

Abbreviations: bi*CEBPA*, biallelic mutation in the CCAAT/enhancer-binding protein alpha; CI, confidence interval; CR, complete remission; mo*CEBPA*, monoallelic mutation in the CCAAT/enhancer-binding protein alpha.


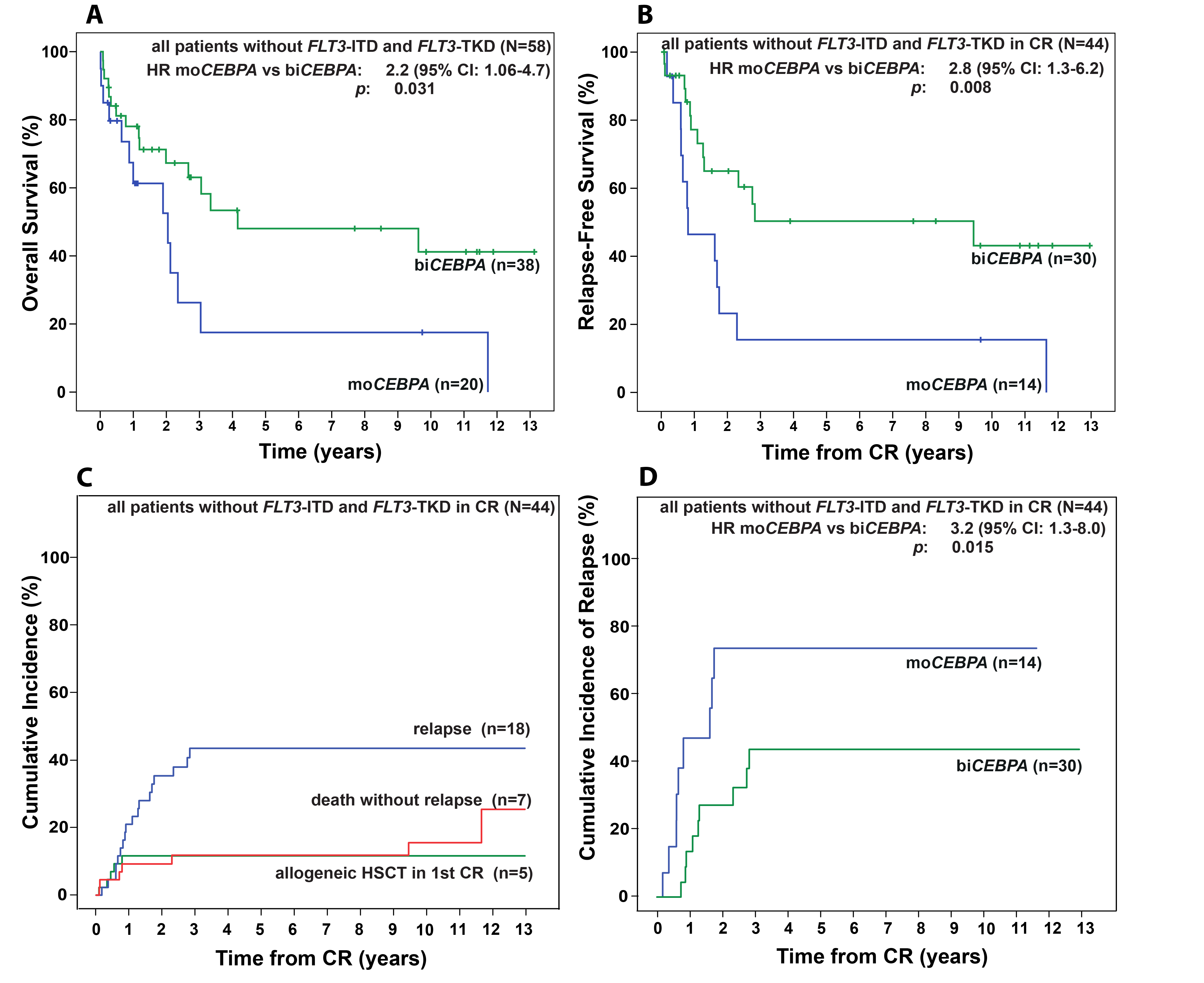

Supplement: Additional file 1: — Data supplement. [file 13045_2014_55_MOESM1_ESM.docx]
